# Supplementary material for: Bhadran’s point of generation segregation theory for behavioral precision in biomedical waste management
Source: Sci Rep. 2025 Dec 16;16:2531. doi: 10.1038/s41598-025-32195-4 (PMC12820305; doi:10.1038/s41598-025-32195-4)
Supplement: Supplementary file 4 — Supplementary Material 4 [file 41598_2025_32195_MOESM4_ESM.docx]

**Bhadran’s Point-of-Generation Segregation Theory (PGST): A Novel Behavioral Framework and Metric System for Biomedical Waste Management**

Renjith Seela Bhadran ^1^, Damodaran Vasudevan ^2^

1. Department of Public Health, Amrita School of Medicine, Amrita Institute of Medical Sceicnes, Amrita Vishwa Vidyapeetham, Kochi, Kerala, India
2. Department of Health Sciences Research, Amrita School of Medicine, Amrita Institute of Medical Sciences, Amrita Vishwa Vidyapeetham, Kochi, Kerala, India

*Corresponding Author: Prof. Damodaran Vasudevan, Dean of Research, Department of Health Sciences Research, Amrita School of Medicine, Amrita Institute of Medical Sceicnes, Amrita Vishwa Vidyapeetham, Kochi, Kerala, India. Email:* [*dmvasudevan@yahoo.co.in*](mailto:dmvasudevan@yahoo.co.in)

**Abstract**

Biomedical waste (BMW) mis‑segregation remains a persistent global challenge, threatening infection control, occupational safety, and environmental sustainability. Recognizing that waste segregation is ultimately a behavioral act, this study conceptually introduces Bhadran’s Point‑of‑Generation Segregation Theory (PGST) — a proposed hybrid behavioral‑systems model designed to measure, benchmark, and improve BMW management. Objective: To conceptualize and operationalize a framework that links micro‑level staff behaviors to institutional waste outcomes and global performance classification. Methodology: PGST integrates six constructs — Segregation Accuracy, Occupational Hazard Risk, Environmental Contamination Potential, Irreversible Contamination Index, Segregation Compliance Behavior, and Training Effectiveness — and is anchored by Moment‑Based Precision Behavioral Fidelity (MBPBF), which quantifies four behavioral elements (Cognitive Anchoring, Visual Discrimination, Repetition Reinforcement, and Error‑Responsive Feedback). Behavioral metrics generate the Precision Behavior Score (PBS), Precision Change Score (PCS), Point‑of‑Generation Segregation Accuracy (PGSA), and Point-of-Generation Segregation Index (PGS Index). While PGSA feeds into the Waste Quality Metrics (WQM) composite and the Global Segregation Safety Scale (GSSS), a five‑tier benchmarking tool from EcoPlatinum to EcoBlack, the PGS Index serves as an internal behavioural diagnostic index Results: Illustrative application of PGST suggests that higher behavioral precision was strongly correlated with segregation accuracy and overall institutional waste quality. The model demonstrates that the Precision Behavior Score (PBS), Precision Change Score (PCS), and Point of Generation Segregation Accuracy (PGSA) reliably predict Waste Quality Metrics (WQM) and placement on the Global Segregation Safety Scale (GSSS). These findings highlight that measuring behavioral fidelity at the point of generation provides actionable insights for training design, compliance audits, and evidence-based policy development, as part of a proposed theoretical framework pending empirical validation.

*Keywords: Biomedical Waste Management, Proposed Behavioral Framework, Point of Generation Segregation Theory (PGST), Moment-Based Precision Behavioral Fidelity (MBPBF), Point of Generation Segregation Accuracy (PGSA), Waste Quality Metrics (WQM), Global Segregation Safety Scale (GSSS), Point of Generation Segregation Index (PGS Index), Waste Segregation Accuracy, Public Health, Occupational Safety*

**Introduction**

Biomedical waste (BMW) management remains a critical challenge for modern healthcare, directly impacting infection control, occupational safety, and environmental sustainability ^1,2^. Despite advances in downstream technologies such as autoclaves, incinerators, and chemical disinfection, these interventions cannot undo errors made at the point of waste generation; once hazardous items are mis-sorted, contamination spreads irreversibly through the system^1-3^. This persistent weakness underscores the need for a framework that directly links individual behavioral decisions to institutional safety and quality outcomes^4-6^.

Bhadran’s Point‑of‑Generation Segregation Theory (PGST) is a proposed conceptual behavioral framework that addresses this gap by reframing waste segregation as fundamentally a behavioral act. It asserts that the accuracy of segregation at the exact moment of waste generation is the single most critical determinant of biomedical waste management quality, safety, and sustainability. PGST introduces a set of theoretical constructs — including the Precision Behavior Score (PBS), Point of Generation Segregation Accuracy (PGSA), Point-of-Generation Segregation Index (PGS Index), Waste Quality Metrics (WQM), and Global Segregation Safety Scale (GSSS) — all of which are presently conceptual and pending empirical validation.

**Literature Review**

Global evidence consistently demonstrates that failures in biomedical waste (BMW) segregation at the source compromise the integrity of the entire waste stream^1,7^. Studies from India^,4,20^, Nigeria ^10^, Ethiopia ^11^, Spain ^5^, and Malaysia ^12^ reveal a recurring pattern: once waste is misclassified at the point of generation, even sophisticated downstream treatments—such as incineration, autoclaving, or chemical disinfection—cannot fully neutralize the associated hazards ^1,2^. These lapses contribute to environmental contamination ^1,3^, needle-stick injuries ^13^, regulatory non-compliance ^7,8^, and substantial financial and material losses ^1,3^.

Although both World Health Organization (WHO)^1,2^ and India’s Central Pollution Control Board (CPCB) ^7^ prescribe strict adherence to source-level segregation using the color-coded bins, existing frameworks rarely establish a clear link between frontline behavioral accuracy and broader institutional safety performance ^4-6^. This gap highlights the need for a theory-driven approach that connects micro-level staff actions during disposal with measurable macro-level outcomes. Bhadran’s Point-of-Generation Segregation Theory (PGST) addresses this need by proposing a behaviorally anchored, quantifiable model for BMW segregation.

**Identification of the Research Gap**

The identification of the research gap emerged through a structured investigative process, initiated by a pilot study conducted at Amrita Hospital, Kochi, Kerala—a facility nationally recognized for excellence in biomedical waste management^14^.

This pilot study revealed not only variations in segregation accuracy across different staff categories but also subtle behavioral inconsistencies that persisted despite the availability of adequate infrastructure and prior training^4,15^. While previous research has examined segregation primarily through the lenses of compliance, awareness, and training effectiveness^1,7^, these studies only indirectly acknowledge the role of behavioral factors. None, however, have developed a dedicated behavioral model to explain these recurring patterns.

Building on this gap, the present study positions biomedical waste segregation as fundamentally a behavioral act—shaped by micro-level habits, precision in execution, attentional focus, and routine decision-making^6,15^—elements that remain underexplored in existing literature. A comprehensive review of prior studies further underscored a critical omission: the absence of conceptual frameworks that examine onsite waste segregation through the lens of behavioral science.

While numerous studies have explored factors such as compliance, awareness, training effectiveness, and policy implementation across diverse contexts—including India^4,20^, Nigeria^10^ , Ethiopia^11^, Spain^5^, Tunisia^6^, and Malaysia^12^—very few, if any, have addressed how micro-behavioral drift, such as momentary lapses, habituated shortcuts, or perceptual oversights, can profoundly compromise systemic outcomes.

This omission is particularly striking given WHO’s repeated emphasis on point-of-generation accuracy as the cornerstone of effective biomedical waste management^1,2^.

Evidence from well-regulated and well-resourced environments demonstrates that behavioral inconsistencies at the point of generation can trigger cascading failures across entire waste streams, undermining even advanced downstream treatments such as incineration, autoclaving, or chemical disinfection^2,16,17^. Yet, despite this recognition, existing research has largely treated segregation as a technical or procedural task rather than a dynamic, behavior-driven process.**This underexplored perspective—viewing segregation as a frontline behavioral act vulnerable to drift and variability—constitutes a significant gap in existing academic scholarship. To address this, the present study proposes a theory-informed, behavior-centric model that positions Precision Segregation Behavior (PSB) at the core of biomedical waste governance. It investigates how subtle variations in PSB influence broader performance indices such as Point-of-Generation Segregation Accuracy (PGSA) and the Point-of-Generation Segregation Index (PGS Index). By reframing segregation through a behavioral lens, this study offers a novel contribution to the intersecting domains of public health, hospital safety, and environmental sustainability^2,4,7,15,16^ .**

**Comparative Construct Analysis and Theoretical Foundation**

Building on the identified research gap, a broader evidence synthesis was undertaken by screening over 50 peer-reviewed publications on point-of-generation segregation and onsite biomedical waste practices. From these, ten key studies were selected for detailed comparative analysis based on construct depth, methodological rigor, and geographic diversity (Table 1). Six critical constructs consistently emerged as determinants of effective biomedical waste management: Training Effectiveness (TE), Segregation Accuracy (SA), Occupational Hazard Risk (OHR), Environmental Contamination Potential (ECP), Irreversible Contamination Index (ICI), and Compliance Behavior (CB). While these constructs may appear operational, comparative review revealed a shared foundation — each is ultimately determined by human action at the precise moment of waste disposal. Training effectiveness only translates into improved compliance if it reshapes habitual disposal behavior (TE → CB) ^4,6,15^; segregation accuracy depends on the simple but critical act of a single hand placing an item in the correct bin (SA) ^1,2,11^; and the cascade of health, safety, and environmental risks (OHR, ECP, ICI) is initiated or prevented in that same instant ^5,7,16,17^.

Countries integrating robust training with real-time monitoring and behavioral reinforcement — such as India (AIIMS Bhubaneswar)^4^, Spain ^5^, and Tunisia^6^ — reported sustained system-wide gains, while those where behavioral integration at the point of generation was absent — such as Uganda^18^ and Italy^17^— showed limited or short-lived impact despite policy frameworks. This construct-based comparison (Table 1) not only maps global practices but crystallizes a conceptual shift: biomedical waste management is fundamentally a behavioral act. This recognition provided the pivotal bridge to conceptualizing the Point-of-Generation Segregation Theory (PGST), reframing segregation as a measurable, behavior-driven system with direct implications for safety, environmental sustainability, and public health governance.

**Framework Derivation and Methodological Basis**

2.1 Data Source and Comparative Matrix Construction

The foundation of Bhadran’s Point-of-Generation Segregation Theory (PGST) was built upon a cross-country comparative synthesis of 50+ peer-reviewed studies on biomedical waste segregation, of which ten were selected based on methodological rigor, construct relevance, and data completeness. Data used in Tables 1 and 2 were directly extracted from published literature and institutional reports, with no simulated or artificially generated values. When quantitative data were incomplete, consistent qualitative descriptors (e.g., “improved,” “declined,” “not assessed”) were retained and standardized through the Positive–Negative–Borderline (PNB) framework for uniform comparison.

2.2 PNB Categorization Logic and Scoring Basis

Each country’s data were coded across six behavioral and operational domains—Training Effectiveness, Segregation Accuracy, Occupational Hazard, Environmental Contamination, Irreversible Contamination, and Compliance Behavior.

- Positive (P) indicates favorable or improving performance (e.g., >80 % segregation accuracy, reduced sharps injuries).
- Negative (N) represents performance gaps (e.g., high error rates, poor bin labeling, or unsafe disposal).
- Borderline (B) denotes mixed or inconclusive results (e.g., self-reported gains without quantitative support).

Each domain was reviewed independently by cross-verifying data trends and consistency across studies. The PNB-coded dataset was then pattern-matched across cases, revealing consistent convergence at the behavioral act of segregation—the foundational construct for PGST.

2.3 Variable and Coefficient Determination (α–ζ Series)

The mathematical representation of the Point-of Generation Segregation Index (PGS Index), developed within the PGST framework, incorporates six normalized coefficients (α–ζ), corresponding to the six behavioral domains. These coefficients —Point-of-Generation Segregation Accuracy (PGSA), Occupational Hazard Risk (OHR), Environmental Contamination Potential (ECP), Intra-facility Containment Integrity (ICI), Staff Compliance Behavior (SCB), and Training & Engagement (TE)—were conceptually derived from the relative frequency and contextual significance of each determinant as reported in the literature. Each coefficient was assigned within a 0–1 theoretical weighting scale to ensure that no single construct dominates the framework. The weights are logical placeholders, intended for empirical calibration through future field-based validation studies.

2.4 PGSA and WQM Integration

The Point-of-Generation Segregation Accuracy (PGSA) index and Waste Quality Metrics (WQM) are mathematically interlinked to represent behavioral precision and outcome quality respectively.

PGSA quantifies segregation precision at the generation point by aggregating weighted behavioral scores derived from PNB-coded variables.

WQM translates these precision scores into a composite waste quality output, where higher PGSA scores yield proportionally improved WQM values.

This logic allows for benchmarking behavioral precision (input) against segregation quality (output), establishing a reproducible analytical pathway within PGST.

2.5 Transparency and Reproducibility

All constructs, variables, and their interrelationships were defined explicitly within the manuscript and summarized in a notation table (α–ζ, PGSA, WQM, PBS, GSSS). This ensures that the conceptual framework remains transparent, internally consistent, and reproducible for future empirical verification.**Table 1. Biomedical Waste Segregation Metrics by Country (Refined Summary)**

| **Biomedical Waste Segregation Metrics** | | | | | | |
| --- | --- | --- | --- | --- | --- | --- |
| **Country** | **Training Effectiveness (TE)** | **Segregation Accuracy (SA)** | **Occupational Hazard RISK (OHR)** | **Environmental Contamination Potential (ECP)** | **Irreversible Contamination Index (ICI)** | **Compliance Behavior (CB)** |
| **India (AIIMS Bhubaneswar)**^4^ | ↓ Segregation Deficiency Index from 1.10% to 0.03% (p < 0.0001) *(Mondal, 2022)* | High post-training *(Mondal, 2022)* | Reduced error-related exposure *(Mondal, 2022)* | Improved post-monitoring *(Mondal, 2022)* | Not assessed | Large reduction in violations *(Mondal, 2022)* |
| **Nigeria**^10^ | Ongoing training recommended *(Awodele et al., 2016)* | ~91.5% self-reported segregation *(Awodele et al., 2016)* | Sharps injuries reported *(Awodele et al., 2016)* | Missorted infectious waste in general bins *(Awodele et al., 2016)* | Not assessed | Self-reported high, but varied *(Awodele et al., 2016)* |
| **Ethiopia (Bale Zone)**^11^ | Onsite bins doubled odds of good segregation *(AOR = 2.10)* *(Sahiledengle, 2019)* | 53.8% self-reported “good” practice *(Sahiledengle, 2019)* | Frequent needlestick injuries *(Sahiledengle, 2019)* | Exposure risk from poor segregation *(Sahiledengle, 2019)* | High rejection of mixed batches *(Sahiledengle, 2019)* | Only ~54% compliance *(Sahiledengle, 2019)* |
| **Spain**^5^ | Post-training intervention reduced waste by 6.2% (p < 0.05) *(Mosquera et al., 2014)* | Statistically significant improvement *(Mosquera et al., 2014)* | Indirect safety benefits *(Mosquera et al., 2014)* | Improved with correct bin use *(Mosquera et al., 2014)* | Not assessed | Increased compliance post-intervention *(Mosquera et al., 2014)* |
| **Tunisia**^6^ | Training increased segregation rates *(Bannour et al., 2024)* | Sharps: 60.3→77.6%, Soft: 32.5→72.4% (p < 0.001) *(Bannour et al., 2024)* | Labeling errors persisted; sharps risk reduced *(Bannour et al., 2024)* | Ongoing labeling and disposal concerns *(Bannour et al., 2024)* | Not assessed | Behavior shift noted post-training *(Bannour et al., 2024)* |
| **Italy**^17^ | Not available *(Amariglio & Depaoli, 2021)* | 57% mis-segregation in OTs *(Amariglio & Depaoli, 2021)* | Cross-contamination due to bin mix-ups *(Amariglio & Depaoli, 2021)* | Recycling loss from misclassified waste *(Amariglio & Depaoli, 2021)* | Not assessed | Low adherence in high-risk areas *(Amariglio & Depaoli, 2021)* |
| **Malaysia**^12^ | Digital tools proposed for training *(Mohamed et al., 2024)* | Manual errors common; tech suggested *(Mohamed et al., 2024)* | Sharps/PPE in wrong bins *(Mohamed et al., 2024)* | Pathogen leakage from poor sorting *(Mohamed et al., 2024)* | Inferred from mishandling *(Mohamed et al., 2024)* | Inconsistent without digital support *(Mohamed et al., 2024)* |
| **Uganda**^18^ | Limited facility-level training *(Mugambe et al., 2012)* | Only ~45% had bins at generation point *(Mugambe et al., 2012)* | High needlestick injuries *(Mugambe et al., 2012)* | Minimal monitoring; risk inferred *(Mugambe et al., 2012)* | Not assessed | Low adherence in labs/wards *(Mugambe et al., 2012)* |
| **Pakistan**^19^ | Targeted training reduced injuries *(Qaiser et al., 2013)* | Improved sharp waste sorting *(Qaiser et al., 2013)* | Sharps exposure reduced after education *(Qaiser et al., 2013)* | Not assessed *(Qaiser et al., 2013)* | Not assessed | Pre-post improvement observed *(Qaiser et al., 2013)* |
| **India (Karnataka – Asadullah)**^20^ | In-house training linked to high knowledge *(Gadicherla et al., 2016)* | Knowledge high; practice inconsistent *(Gadicherla et al., 2016)* | Not assessed  *(Gadicherla et al., 2016)* | Not assessed *(Gadicherla et al., 2016)* | Not assessed | Good theoretical compliance; varied behavior *(Gadicherla et al., 2016)* |

The data in Table 1 reveal a clear global pattern. Facilities with strong training programs and active monitoring mechanisms—notably India (AIIMS Bhubaneswar) ^4^, Spain ^5^, and Tunisia^6^—consistently achieved measurable improvements in segregation accuracy, compliance behavior, and reductions in occupational hazards. In contrast, countries with weak source‑level integration or minimal facility‑level training, such as Uganda and Italy, showed persistent mis‑segregation, heightened contamination risks, and fragmented system outcomes. These findings underscore that infrastructure alone is insufficient; the decisive factor is behavior at the point of generation. This evidence strengthens the case for behaviorally anchored frameworks like PGST, which explicitly link staff training, behavioral precision, and real‑time decision‑making to institutional safety, regulatory compliance, and long‑term sustainability.

**PNB Categorization**

Given the diverse and non‑uniform nature of biomedical waste segregation data across countries, this study introduces an original evaluative tool—Bhadran’s PNB Categorization. This framework represents a novel contribution of the research and does not draw from any pre‑existing model in academic literature. It was specifically designed for use in comparative public health and waste management assessments, particularly in contexts where quantitative indicators are inconsistent, unavailable, or fragmented (Table 2). The categorization system classifies findings into three intuitive categories: **Positive** (P) for favorable or improving performance, **Negative** (N) for suboptimal or concerning outcomes, and **Borderline** (B) for mixed, inconclusive, or unassessed data. Developed to underpin the cross‑country comparative matrix, Bhadran’s PNB method enables pattern recognition, benchmarking, and theory‑building across diverse healthcare systems. By mapping systemic strengths and weaknesses—especially at the point of waste generation—it offers a practical, transparent tool that informs both research and policy, providing actionable insights for interventions and training strategies.

**Table 2 : Comparative Matrix- PNB (Positive–Negative–Borderline) Format**

| **Comparative Matrix: Biomedical Waste Segregation Metrics by Country (PNB** (Positive–Negative–Borderline) **Format)** | | | | | | |
| --- | --- | --- | --- | --- | --- | --- |
| **Country** | **Training Effectiveness** | **Segregation Accuracy** | **Occupational Hazard** | **Environmental Contamination Potential** | **Irreversible Contamination** | **Compliance Behavior** |
| **India (AIIMS Bhubaneswar)^4^** | **P** – Significant improvement post-training | **P** – High accuracy | **P** – Reduced exposure | **P** – Post-monitoring improved | **B** – Not assessed | **P** – Large violation drop |
| **Nigeria^10^** | **P** – Ongoing training recommended | **P** – High self-report (91.5%) | **N** – Sharps injuries | **N** – Missorting seen | **B** – Not assessed | **B** – Varied self-report |
| **Ethiopia^11^ (Bale Zone)** | **P** – Bins linked to 2× better odds | **N** – Only 53.8% good practice | **N** – Frequent injuries | **N** – Risk from segregation failure | **P** – High rejection of mixed batches | **N** – ~54% compliance |
| **Spain^5^** | **P** – Reduced waste 6.2% post-training | **P** – Statistically improved | **P** – Safety benefits | **P** – Bin usage improved outcomes | **B** – Not reported | **P** – Post-training rise |
| **Tunisia^6^** | **P** – Sharps & soft waste improved | **P** – Sharps: 60.3→77.6%, Soft: 32.5→72.4% | **N** – Labeling errors remain | **N** – Disposal concerns remain | **B** – Not reported | **P** – Behavior shift seen |
| **Italy^17^** | **B** – Not available | **N** – 57% mis-segregation in OTs | **N** – Cross-contamination | **N** – Recycling loss | **B** – Not assessed | **N** – Low adherence |
| **Malaysia^12^** | **P** – Digital tools proposed | **N** – Manual errors common | **N** – Sharps/PPE misplacement | **N** – Pathogen leakage | **N** – Mishandling inferred | **N** – Inconsistent compliance |
| **Uganda^18^** | **N** – Limited facility training | **N** – Only ~45% bin access | **N** – High needlestick injuries | **N** – Monitoring minimal | **B** – Not documented | **N** – Low adherence |
| **Pakistan^19^** | **P** – Training reduced injuries | **P** – Sharp sorting improved | **P** – Less exposure post-education | **B** – Not explored | **B** – Not discussed | **P** – Pre-post behavior better |
| **India (Karnataka) ^20^** | **P** – High knowledge via in-house training | **N** – Practice inconsistent | **B** – Not emphasized | **B** – Not emphasized | **B** – Not assessed | **B** – Theory good; practice varied |

The PNB categorization in Table 2 maps how different countries align across six core biomedical waste domains: training effectiveness, segregation accuracy, occupational hazard, environmental contamination potential, irreversible contamination, and compliance behavior. Data were drawn from peer-reviewed literature, institutional reports, and national audits, with each domain rated using a three-tier scale — Positive (P) for clear, consistent, or improving outcomes (e.g., >80 % segregation accuracy, reduced injury rates, or demonstrably successful training programs); Negative (N) for serious gaps or poor performance (e.g., frequent needle-stick injuries, recurrent mis-segregation, or absence of effective interventions); and Borderline (B) for mixed, uncertain, or partial results (e.g., pilot projects, self-reported improvements, or inconsistent practices). Ratings were assigned using uniform criteria and supported by documented justification to ensure transparency, reliability, and comparability.

India (AIIMS Bhubaneswar) ⁴ and Spain stand out with predominantly Positive (P) scores, reflecting robust training systems, high segregation accuracy, and consistent compliance. Pakistan ¹⁹ also performs well, whereas Tunisia ⁶ shows marked progress yet records Negative (N) ratings for Occupational Hazard and Environmental Contamination due to labeling and disposal deficiencies. At the other extreme, Uganda ¹⁸ and Malaysia ¹² present mostly Negative (N) ratings, revealing systemic vulnerabilities and unsafe handling practices. Several nations — including Italy and India (Karnataka) ²⁰ — occupy a Borderline (B) category on multiple metrics, indicating partial success but inconsistent application.

By consolidating fragmented, heterogeneous evidence into a standardized P–N–B framework, Bhadran’s PGST advances both practical benchmarking and theoretical understanding by framing biomedical waste management as a behavioral act shaped by training quality, compliance culture, and risk perception. This behavioral perspective equips policymakers to design interventions that address not only infrastructure and policy gaps but also the human factors that determine segregation accuracy and safe handling outcomes.

To ensure that the PNB Categorization operated not merely as a descriptive tool but as an analytical bridge, each coded construct (Training Effectiveness, Segregation Accuracy, etc.) was subjected to cross-case pattern matching across the ten-country dataset. This comparative synthesis followed a grounded analytical logic similar to qualitative comparative analysis (QCA), allowing recurrent behavioral determinants to emerge inductively from the evidence. Patterns consistently showed that high performance across all six constructs converged at one empirical point — the precise behavioral act of segregation at waste generation. This convergence, validated through repeated coding and matrix comparison, formed the analytical foundation for the Point-of-Generation Segregation Theory (PGST). Hence, PGST was not an abstract conceptualization but a grounded theoretical outcome derived through systematic pattern recognition within the PNB-coded comparative dataset.

**Theory Development**

**PNB Framework and Evolution Toward PGST** — To enable meaningful cross-country comparison of biomedical waste (BMW) segregation practices despite highly variable reporting formats, this study developed the **PNB Categorization** as a structured qualitative coding system. Drawing from diverse sources — peer-reviewed studies, institutional audits, and policy reports ^1,2,3,4,5,11,16,18,20^ — PNB synthesized information across six domains: *Training Effectiveness*, *Segregation Accuracy*, *Occupational Hazard*, *Environmental Contamination*, *Irreversible Contamination*, and *Compliance Behavior*. While structurally aligned with global tools such as the WHO WASH FIT evaluation framework^21^ and the WHO’s healthcare waste categorization^6^, PNB is distinctive in being both source-specific and prescriptive, targeting performance at the exact point of waste generation — a critical gap that existing models do not fully address ^1,4,11,15^.

Insights from this PNB-based comparative analysis revealed a stark divide: institutions with robust training and infrastructure (e.g., India (AIIMS Bhubaneswar)& Spain) consistently achieved high segregation accuracy^4^,^5^, whereas low-resource settings (e.g., Uganda, Ethiopia) experienced frequent contamination events^11,18^. This evidence confirmed a universal failure point — contamination initiated at the source is rarely reversible downstream^1,2,22^. Recognizing this, the framework naturally progressed toward a **performance grading construct** that could not only compare institutions but also reflect the *behavioral disposition* influencing segregation practices ^7,13,16,17^, holding potential to serve as a unified measure linking operational efficiency with institutional culture in biomedical waste management ^2,3,8,10^.

**Conceptual Roots, Habitual Precision, and Precision Behavior**

The conceptual foundations of the Point-of-Generation Segregation Theory (PGST) draw upon both modern behavioral science and Kerala’s illustrious mathematical heritage. Central inspiration comes from Mādhava of Saṅgamagrāma (c. 1340–1425), founder of the Kerala School of Mathematics, and Jyesthadeva (c. 1530), whose Yuktibhāṣā (translated as Ganita-Yukti-Bhāṣā or Rationales in Mathematical Astronomy) translated highly abstract mathematical reasoning into clear, stepwise algorithms^23^. This intellectual lineage exemplifies yukti—reasoned explanation by which complex ideas are broken into precise, teachable steps without loss of rigor^23^. Modern editions, with English translation and commentary published by Springer, preserve the Kerala School’s systematic approach to logic and computation.

From this tradition, two complementary principles inform PGST. Mādhava’s work embodies precision behavior—the application of exact reasoning at each decisive step^24,25^. Jyesthadeva’s Yuktibhāṣā embodies habitual precision—the disciplined, iterative refinement of results through consistent, exacting practice^23,25^. PGST fuses these principles: precision behavior ensures flawless execution at the critical moment of waste disposal, while habitual precision ensures that segregation accuracy is maintained consistently over time, even under variable conditions. Together, they transform biomedical waste segregation from a procedural requirement into a deeply embedded safety culture.

At the operational level, PGST rests on three interconnected pillars:

- Moment Alignment – acting immediately at the point of waste generation.
- Micro Accuracy – flawless identification and disposal of every item.
- Reinforced Reflex – repetition so consistent that correct action becomes automatic, even under stress.

These pillars mirror the Kerala mathematicians’ disciplined, stepwise methodology, ensuring that countless small, precise actions accumulate into reliable, system-level safety. By combining historical insight, behavioral theory, and practical rigor, PGST establishes a framework where every micro-level action contributes to maximal occupational safety and environmental protection.

**PGST Definition and Scope**

**Definition**

Bhadran’s Point of Generation Segregation Theory (PGST) is defined as:

“*An integrated public health systems model that identifies segregation accuracy at the exact point and moment of waste generation as the single most critical determinant of biomedical waste management quality, framing every disposal act as a behavioral decision whose cumulative precision shapes institutional safety, compliance, and environmental performance.*”

**Theory Statement**

PGST posits that errors committed at the exact point of biomedical waste generation^26,27^ initiate a chain of irreversible contamination, occupational risk^28^, and systemic inefficiency^22^—undermining safety, compliance, and environmental sustainability across the entire waste management continuum^1^. Conversely, when segregation is executed with precision at the source^26,28^ , it sets off a positive cascade: reinforcing moment-level behavioral fidelity among staff, elevating institutional segregation accuracy^22^, and enabling healthcare facilities to ascend global safety and quality tiers^1^. PGST reframes biomedical waste segregation not merely as a regulatory requirement but as a behaviorally governed systems function, where every single disposal act at the bin holds the potential to either preserve or compromise the entire ecosystem. This dual framing—behavioral at the micro level, systemic at the macro level—establishes PGST as a foundational theory for advancing global biomedical waste safety standards.

**Behavioral Drift and the Precision Imperative in Biomedical Waste Segregation**

The Point of Generation Segregation Theory (PGST) asserts that accurate biomedical waste segregation at the source is not merely a procedural act but a behavioral responsibility that underpins the safety and sustainability of the entire hospital waste management system. A major threat to this is what is here termed **behavioral drift**—the subtle, progressive deviation from correct practices caused by factors such as habituation, fatigue, or diminished vigilance in clinical settings. Unlike outright negligence, behavioral drift is insidious, often escaping detection while quietly eroding precision over time.

This phenomenon finds philosophical resonance in the mathematical legacy of Sangamagrāma Mādhava (c. 1340–1425), founder of the Kerala School of astronomy and mathematics, who pioneered infinite series—particularly those for π—with remarkable precision through corrective terms that anticipated later analytic methods by centuries^29^. His work reveals how even infinitesimal deviations or incomplete terms can compound into notable discrepancies—a principle mirrored in behavioral precision: small, repeated lapses can accumulate into significant systemic risk. Just as Mādhava’s emphasis on exactness ensured mathematical reliability, Bhadran’s PGST underscores the necessity of consistent behavioral precision in biomedical waste segregation. Addressing behavioral drift through systematic reinforcement, training, and a culture of precision is thus essential to preserving integrity at the point of generation.

**PGST Scope and Framework:**

Rooted in the guidelines of the World Health Organization (WHO) and Central Pollution Control Board (CPCB) of India, Bhadran’s PGST links source‑level accountability with measurable institutional outcomes. Each frontline worker — nurse, technician, or doctor — is assessed using a Precision Behavior Score (PBS) derived from four behavioral elements: Cognitive Anchoring, Visual Discrimination, Repetition Reinforcement, and Error‑Responsive Feedback. These PBS scores are aggregated into the Point of Generation Segregation Accuracy (PGSA), which becomes the central metric of PGST.

From PGSA, two interconnected pathways emerge under the Bhadran’s PGST umbrella:

- **Operational stream:** PGSA feeds into the Waste Quality Metrics (WQM) system and ultimately maps onto the Global Segregation Safety Scale (GSSS), producing structured measures for auditing, benchmarking, and policy alignment.
- **Behavioral stream:** PGSA informs the Point of Generation Segregation Index (PGS Index), a composite indicator that reflects the precision culture and behavioral integrity within a facility.

**Why PGST Matters:**

Bhadran’s PGST underscores that all operations are ultimately behavioral acts. Individual behaviors and segregation accuracy scores roll up into PGSA, which captures the collective behavioral pattern of the institution. This aggregation converts thousands of split‑second micro‑decisions into a single institutional score, demonstrating that system performance is simply the sum of consistent behaviors. PGST reframes biomedical waste management not merely as a compliance task, but as a behavior‑anchored system of precision that can be measured, improved, and scaled globally.

**From Individuals to PGST Core Constructs**

Bhadran’s PGST model operates as a structured continuum, transforming individual micro behaviors into institutional benchmarks and ultimately global classifications. At its foundation lies the Moment-Based Precision Behavioral Fidelity (MBPBF) framework, which evaluates four micro actions — Cognitive Anchoring (CA), Visual Discrimination (VD), Repetition Reinforcement (RR), and Error Responsive Feedback (ERF) — to assess each worker’s segregation behavior.

Each micro-action is scored on a normalized 0 – 1 scale (0 = incorrect behavior; 1 = accurate behavior) during real-time observation.

The four values are then consolidated into a composite indicator for each staff member, termed the Precision Behavior Score (PBS).

Equation (1): PBS = (CA + VD + RR + ERF)/4

The aggregation of all PBS values across staff yields the Point-of-Generation Segregation Accuracy (PGSA), representing the hospital’s overall segregation accuracy.

Equation (2): PGSA = Σ PBSᵢ / N,

where PBSᵢ = Precision Behavior Score of staff member i, and N = total number of staff observed.

Example: If 10 staff members record an average PBS of 0.82, then PGSA = 0.82 → High Institutional Accuracy (≥ 0.80).

This PGSA then integrates into the Point-of-Generation Segregation Index (PGS Index), comprising six interconnected pillars: Segregation Accuracy (SA), Occupational Hazard Risk (OHR), Environmental Contamination Potential (ECP), Irreversible Contamination Index (ICI), Segregation Compliance Behavior (SCB), and Training Effectiveness (TE), with SA serving as the anchor construct influencing all others.

**Behavioral Engine: MBPBF**

The behavioral engine of Bhadran’s PGST is Moment-Based Precision Behavioral Fidelity (MBPBF), a real-time observation system that captures the accuracy of staff in segregating biomedical waste at the exact disposal moment. It operationalizes the four micro-actions (CA, VD, RR, ERF) defined earlier, converting them into measurable indicators of behavioral precision and reliability in practice.

These four components produce the Precision Behavior Score (PBS) for each staff member, , as shown above, and enable longitudinal assessment through the Precision Change Score (PCS), which measures improvement from interventions:

Equation (3): PCS = PBS (Post) – PBS (Baseline)

**Point of Generation Segregation Accuracy** (PGSA)

Point-of-Generation Segregation Accuracy (PGSA) is the institutional average of all individual PBS values, calculated as in Equation (2). PGSA serves as the gateway metric within the PGS Index, uniquely linking micro-level behavioral observations to organizational-level quantitative measures.

Its range (0 – 1) corresponds to Low (< 0.60), Moderate (0.60 – 0.79), and High (≥ 0.80) accuracy categories. This dual-pathway structure connects operational efficiency (via WQM and GSSS) and behavioral excellence (via PGS Index), an approach rarely addressed in biomedical-waste segregation studies.

The conceptual link underlying the newly proposed PGSA metric originates within Bhadran’s PGST framework and is presented here as a conceptual, yet-to-be-validated analytical bridge in waste-segregation performance evaluation.

Table 3 : Notation Summary for PGST Behavioral Constructs and Derived Metrics

| **Symbol** | **Full Form / Meaning** | **Range / Unit** | **Notes** |
| --- | --- | --- | --- |
| CA | Cognitive Anchoring | 0 – 1 | Correct color-bin identification accuracy |
| VD | Visual Discrimination | 0 – 1 | Ability to differentiate similar waste types |
| RR | Repetition Reinforcement | 0 – 1 | Consistency of correct segregation over trials |
| ERF | Error Responsive Feedback | 0 – 1 | Speed and accuracy of error correction |
| PBS | Precision Behavior Score | 0 – 1 | Mean of CA, VD, RR, ERF for individual staff |
| PCS | Precision Change Score | –1 to +1 | Improvement post-training (positive = better) |
| PGSA | Point-of-Generation Segregation Accuracy | 0 – 1 | Institutional average of PBS |

**Significance of MBPBF**

MBPBF itself is not a score — it is the framework that generates PBS values, which then scale up to PGSA. Together, PBS, PCS, and PGSA provide both instant behavioral snapshots and long‑term improvement trends. This scaling process converts individual behaviors at the bin into institution‑wide accuracy metrics, powering Point of Generation Segregation Theory (PGST) system of waste-quality measurement and safety classification.

**From Constructs to Waste Quality Metrics (WQM)**

The Waste Quality Metrics (WQM) system forms the operational backbone of Bhadran’s PGST, translating behavioral precision and institutional practices into quantifiable performance indicators. It integrates six components—one derived from behavioral fidelity at the source (WQM 1: Point of Generation Segregation Accuracy) and five derived from institutional audits (WQM 2–6: PPE compliance, Spill management, Documentation & Reporting, Temporary Storage safety, and Training & Awareness Maintenance). Together, these generate a composite 100-point score that reflects both behavioral and systemic quality. WQM 1 serves as the behavioral anchor, directly linking micro-level staff actions to institutional outcomes, while WQM 2–6 ensure that operational safeguards and organizational culture are equally accounted for. The resulting WQM score can also be directly mapped to the Global Segregation Safety Scale (GSSS), providing a standardized global benchmark for biomedical waste segregation safety.

**Formulas for Each WQM Component**

1️⃣ **WQM 1** – **Segregation Accuracy Score**

Derived directly from PGSA:

**Equation 4: WQM 1= [ PGSA / 10 ] × 20**

What it measures:

WQM 1 anchors the entire WQM system by translating Point of Generation Segregation Accuracy (PGSA) — the average behavioral precision score — into a 20‑point contribution. This conversion (÷10 ×20) normalizes the 0–10 PGSA scale to the 20-point WQM framework, maintaining equal weightage among all metrics. This shows how accurate staff are in segregating waste at the source.

*Example: PGSA = 7.5 (on a 10‑point scale):*

*WQM 1 = [ 7.5 / 10 ] × 20 = 15 points.*

2️⃣ **WQM 2** – **PPE Compliance**

**Equation 5: WQM 2= [Observed PPE Compliance (%) / 100] ×20**

What it measures:

Whether staff use gloves, masks, gowns, and other PPE consistently while handling biomedical waste.

*Example : PPE adherence = 90%*

*WQM 2 = [ 90 / 100 ] × 20 = 18 points.*

3️⃣ **WQM 3** – **Spill Management**

**Equation 6: WQM 3= [Spill Response Effectiveness (%) /100] × 20**

What it measures:

Evaluates how efficiently spills are contained, cleaned, and reported.

*Example : Spill response effectiveness = 80%*

*WQM 3 = [ 80 / 100 ] × 20 = 16 points.*

4️⃣ **WQM 4** – **Documentation & Reporting**

**Equation 7: WQM 4= [ Documentation Accuracy (%) / 100 ] ×10**

What it measures:

Evaluates completeness of BMW records, logbooks, and incident reports.

Example: Documentation accuracy = 95%

WQM  4 = 95 / 100 × 10 = 9.5 points

5️⃣ **WQM 5** – **Temporary Storage Safety**

**Equation 8: WQM 5= [ Storage Safety Compliance (%) / 100 ] × 10**

What it measures:

Evaluates containment integrity in temporary storage areas (e.g., segregation maintained, lids secured, no leaks).

Example: Storage safety compliance = 70%

WQM  5 = [ 70 / 100 ] × 10 = 7 points.

6️⃣ **WQM 6** – **Training & Awareness Maintenance**

.

**Equation 9: WQM 6= [Training Effectiveness (%) / 100 ] ×20**

What it measures:

Evaluates ongoing training, refresher courses, and application of learning in practice.

Example:

Training effectiveness = 95%

WQM 6 = [ 95 / 100 ] × 20 =19 points.

**Total WQM Calculation**

**Equation 10: WQM Total=WQM 1+WQM 2+WQM 3+WQM 4+WQM 5+WQM 6**

**Substituting numeric values:**

**WQM Total =15+18+16+9.5+7+19=84.5 points (out of 100)**

**Global Segregation Safety Scale (GSSS)**

**Definition:** The Global Segregation Safety Scale (GSSS) is a five-tier international classification system (EcoPlatinum to EcoBlack) that benchmarks the biomedical waste management performance of healthcare institutions. Derived from the total Waste Quality Metrics (WQM) score, GSSS translates operational accuracy into a globally comparable safety tier, enabling consistent assessment across diverse systems and geographies.

**GSSS Calculation**

**MBPBF ➡️ PGSA ➡️ WQM 1 + WQM 2–6 ➡️ Total WQM Score ➡️ GSSS Tier**

The GSSS calculation begins with the Moment-Based Precision Behavioral Fidelity (MBPBF), which assesses four micro-behaviors during waste segregation to generate individual Precision Behavior Scores (PBS). These are averaged to produce the Point-of-Generation Segregation Accuracy (PGSA). From PGSA, a single behavioral metric—WQM 1 (Segregation Accuracy)—is computed using the formula (PGSA ÷ 10) × 20, quantifying behavioral precision. The remaining five indicators—WQM 2 to WQM 6—evaluate PPE compliance, spill management, documentation and reporting, temporary storage safety, and training & awareness. All six WQM scores (each weighted equally at 20 points) are then summed to yield the Total WQM Score (out of 100). This composite score is subsequently mapped onto the Global Segregation Safety Scale (GSSS), a five-tier classification system that translates operational performance into globally recognizable safety tiers (Figure 1).

**Figure 1: GSSS Tiers**


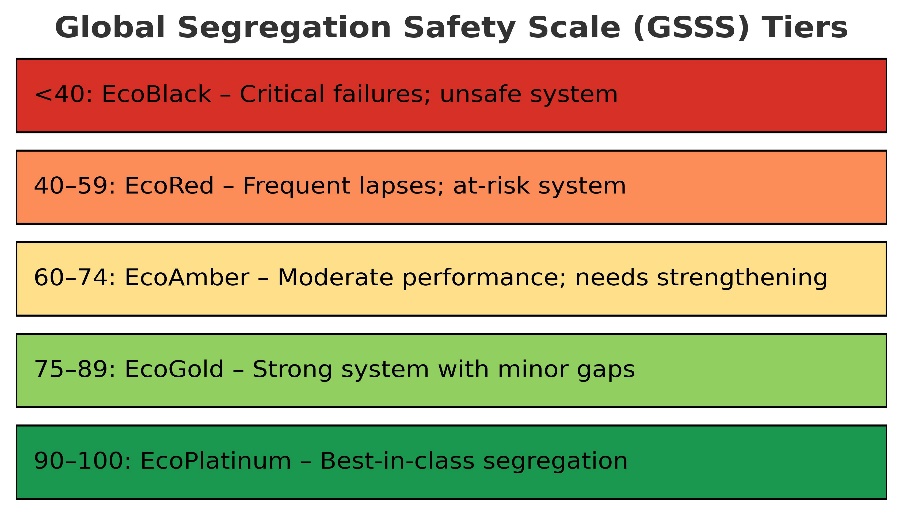


This mapping provides a clear, standardized framework for benchmarking facilities across geographies, directly aligning with PGST’s goal of linking micro-level behavioral precision to macro-level institutional classification.

Based on the example total WQM score calculation:

15 (WQM 1) + 18 (WQM 2) + 16 (WQM 3) + 9.5 (WQM 4) + 7 (WQM 5) + 19 (WQM 6) = **84.5 (out of 100)**

This facility falls into the following GSSS Tier: **EcoGold** (Strong system with minor gaps**)**

**Significance of GSSS in the PGST**

Under the Bhadran’s PGST, GSSS serves as the final institutional classification outcome that reflects the operational quality of biomedical waste segregation at a healthcare facility. While PGST focuses on the micro-level behavioral decisions (via MBPBF and PGSA), GSSS captures the macro-level performance outcome, making it a vital tool for:

- Regulatory audits and safety inspections
- Benchmarking institutional progress over time
- Identifying training needs and process gaps
- Policy alignment and standardization across regions
- Recognition of high-performing institutions

By integrating Moment-Based Precision Behavioral Fidelity (MBPBF), segregation accuracy (PGSA), and operational standards (WQM), GSSS converts granular behavioral data into a universal scorecard. This ensures that every act of precise segregation contributes to institutional advancement and global public health sustainability.

**Linkages Between Operational and Behavioral Streams**

The dual outputs in Bhadran’s PGST emerge through two distinct yet interconnected process routes (Table 4). On the operational stream, inputs move from MBPBF through PBS, PGSA, and WQM to culminate in the GSSS Classification, which enables external benchmarking, ensures regulatory compliance, and situates the institution within global safety tiers. In parallel, the behavioral stream flows from MBPBF through PBS, PGSA, and Core Constructs to yield the PGS Index Score, a diagnostic tool that captures staff behavior patterns, supports internal analysis, and guides targeted training. Together, these parallel linkages ensure that PGST addresses both systemic efficiency and human performance.

**Table 4: Duel Outputs in Bhadran’s PGST**

| **Summary of Dual Outputs in PGST** | | | |
| --- | --- | --- | --- |
| **Stream** | **Process Route** | **Final Output** | **Primary Use** |
| **Operational** | MBPBF → PBS → PGSA → WQM → GSSS | **GSSS Classification** | External benchmarking, regulatory compliance, and global safety tiering |
| **Behavioral** | MBPBF → PBS → PGSA → Core Constructs → PGS Index | **PGS Index Score** | Internal diagnostics, staff behavior analysis, and targeted training |

**Point of Generation Segregation Index (PGS Index): A Composite Score of Elite Precision in Biomedical Waste Segregation**

The PGS Index is a purely behavioral and final composite metric within the Point-of-Generation Segregation Theory framework that quantifies the precision and integrity of waste segregation behavior at the source. Unlike traditional outcome-based audits, it focuses on human actions—how individuals comply with protocols, perceive risks, and apply training during waste segregation. By integrating six key behavioral constructs into a single weighted score, the index captures the cumulative effect of micro-level decisions on occupational safety, environmental contamination potential, and regulatory compliance (Table 5). This behavioral lens makes the PGS Index a vital diagnostic tool to understand and improve segregation practices in real time.

**Equation (11): PGS Index=(α×PGSA)+(β×OHR)+(γ×ECP)+(δ×ICI)+(ε×SCB)+(ζ×TE)**

**where α–ζ are dimensionless weighting coefficients (0 < α–ζ ≤ 1, Σ = 1). All construct values are expressed on a 0–100 scale (%).**

**Table 5: PGS Index Categories**

| **Score Range** | **Category** | **Interpretation** |
| --- | --- | --- |
| 90–100 | **Elite Precision** | Outstanding segregation and safety practices across all domains. Benchmark for excellence. |
| 75–89.99 | **Excellent** | High compliance with minimal risks; minor improvements needed for elite performance. |
| 55–74.99 | **Very Good** *(Needs Improvement)* | Adequate systems in place; moderate inconsistencies exist that need attention. |
| 45–54.99 | **Good** *(Needs Improvement)* | Basic systems functioning, but notable weaknesses in accuracy, safety, or compliance require improvement. |
| < 45 | **Poor** | Minimum standards met, but significant deficiencies in accuracy, safety, or staff behavior demand urgent action. |

**Elite Precision – The Behavioral Gold Standard**

The PGS Index, developed by Renjith Seela Bhadran, represents a behavioral gold standard where **Elite Precision** is not limited to procedural compliance but reflects instinctive and internalized accuracy at the point of segregation. At its core lies Point-of-Generation Segregation Accuracy (PGSA), the central construct that captures the precision of depositing waste into the correct bins at the moment of discard. Surrounding this foundation are five interlinked domains—Occupational Hazard Risk (OHR), Environmental Contamination Potential (ECP), Intra-facility Containment Integrity (ICI), Staff Compliance Behavior (SCB), and Training and Engagement (TE)—which collectively reinforce safety, ecological integrity, systemic containment, behavioral fidelity, and learning effectiveness. Structured on a 100-point scale, these interconnected constructs operate as a unified framework to diagnose not just operational performance but also subtle deviations in practice. In doing so, the PGS Index makes visible what Point-of-Generation Segregation Theory (PGST) terms **behavioral drift**, the small yet significant lapses that arise despite intact infrastructure and protocols, often linked to cognitive fatigue or desensitization.

**Table 6: PGS Index Scoring Formulas Table**

| **PGST Scoring Formulas Table** | | | | |
| --- | --- | --- | --- | --- |
| *(With Purpose & Example for Each Construct — Total Score out of 100)* | | | | |
| **#** | **Construct** | **Formula** | **Purpose** | **Example Calculation** |
| 1 | **PGSA** (Point-of-Generation Segregation Accuracy) | **PGSA = (∑PBSᵢ / N) × 100** | Evaluates bin-level segregation quality at each generation point across the hospital | ((85 + 90 + 100 + 80 + 95) / 5) × 100 = 90 |
|  |  | *(PBSᵢ = individual bin score out of 100)* |  |  |
| 2 | **OHR** (Occupational Hazard Risk) | **OHR = 100 − ((Injuries × 20 + PPE Violations × 10) / Total Staff Observed)** | Assesses threat to healthcare staff from physical injuries and protective protocol violations | 100 − ((2×20 + 4×10) / 30) = 97.33 |
| 3 | **ECP** (Environmental Contamination Potential) | **ECP = 100 − ((Spills × 10 + Unlabelled Bags × 5 + Overflowing Bins × 15) / Audit Units)** | Measures environmental risks due to visible hazards or waste mismanagement | 100 − ((2×10 + 3×5 + 1×15) / 25) = 98 |
| 4 | **ICI** (Intra-facility Containment Integrity) | **ICI = (Secure Transports + Sealed Temp Units) / Total Units Observed × 100** | Examines physical containment during intra-facility transport and temporary storage | (18 / 20) × 100 = 90 |
| 5 | **SCB** (Staff Compliance Behavior) | **SCB = (Staff following all 3 behavioral norms / Total Staff Audited) × 100** | Evaluates behavioral adherence to infection control and segregation norms | (25 / 30) × 100 = 83.33 |
| 6 | **TE** (Training & Engagement) | **TE = (Staff Trained in 6 Months + Passed Post-Tests) / Total Staff × 100** | Tracks recent training coverage and post-training knowledge retention among staff | ((20 + 10) / 40) × 100 = 75 |

PGS Index Formula (Behavioral Weights Model)

**PGS Index=(α×PGSA)+(β×OHR)+(γ×ECP)+(δ×ICI)+(ε×SCB)+(ζ×TE)**

Where:

- α = 0.40 (PGSA – most critical, real-time behavior)
- β = 0.15 (OHR – direct risk prevention)
- γ = 0.15 (ECP – environment-related risks)
- δ = 0.10 (ICI – infrastructure integrity)
- ε = 0.10 (SCB – procedural adherence)
- ζ = 0.10 (TE – long-term behavior shaping through training)

Total sum of weights = 1.00

**Table 7: PGS Index Example Calculation**

| **Construct** | **Value** | **Weight** | **Weighted Score** |
| --- | --- | --- | --- |
| **PGSA** | 90 | 0.4 | 0.40 × 90 = **36.0** |
| **OHR** | 97.33 | 0.15 | 0.15 × 97.33 = **14.6** |
| **ECP** | 98 | 0.15 | 0.15 × 98 = **14.7** |
| **ICI** | 90 | 0.1 | 0.10 × 90 = **9.0** |
| **SCB** | 83.33 | 0.1 | 0.10 × 83.33 = **8.33** |
| **TE** | 75 | 0.1 | 0.10 × 75 = **7.5** |

Total = 36.00 + 14.60 + 14.70 + 9.00 + 8.33 + 7.50 = 90.13

Score**: 90.13 out of 100 → Performance Level: ⭐ Elite Precision**

Table 8. Notation and Symbol Definitions for the PGS Index Formulas

| **Symbol** | **Parameter / Construct** | **Meaning / Definition** | **Range / Unit** | **Source / Derivation** |
| --- | --- | --- | --- | --- |
| **α** | Weight for PGSA | Relative importance of segregation accuracy | 0–1 (dimensionless) | Assigned (behavioral weighting model) |
| **β** | Weight for OHR | Relative importance of occupational hazard risk | 0–1 | Assigned |
| **γ** | Weight for ECP | Relative importance of environmental contamination potential | 0–1 | Assigned |
| **δ** | Weight for ICI | Relative importance of intra-facility containment integrity | 0–1 | Assigned |
| **ε** | Weight for SCB | Relative importance of staff compliance behavior | 0–1 | Assigned |
| **ζ** | Weight for TE | Relative importance of training and engagement | 0–1 | Assigned |
| **PGSA** | Point-of-Generation Segregation Accuracy | Institutional mean segregation accuracy | 0–100 (%) | Derived from PBS aggregation |
| **OHR** | Occupational Hazard Risk | Risk-adjusted score | 0–100 (%) | Computed from injury/PPE data |
| **ECP** | Environmental Contamination Potential | Environmental exposure likelihood | 0–100 (%) | Derived from audit data |
| **ICI** | Intra-facility Containment Integrity | Containment efficiency | 0–100 (%) | Computed from facility logs |
| **SCB** | Staff Compliance Behavior | Norm adherence rate | 0–100 (%) | Observation checklist |
| **TE** | Training & Engagement | Training coverage and retention | 0–100 (%) | Training records |
| **PGS Index** | Composite behavioral index | Weighted sum of six constructs | 0–100 (score) | Final behavioral precision metric |

The PGS Index reframes biomedical waste segregation as a behavioral act rather than a purely operational task. By integrating constructs such as compliance, training, and risk perception, it captures both precision behavior (moment-level accuracy) and habitual behavior (ingrained practice). More than a performance metric, it reflects institutional culture and discipline, serving as a transformative healthcare quality indicator that advances accountability, safety, and sustainable segregation practices.

**Discussion**

The core argument that underpins the Point-of-Generation Segregation Index (PGS Index) developed by Renjith Seela Bhadran is that biomedical waste (BMW) segregation is fundamentally a behavioral act—not merely an operational or system-driven process. While most existing indices assess compliance at a systemic or procedural level ^30,31^, the PGS Index ventures further to examine moment-to-moment behavioral precision and the habitual fidelity demonstrated by individuals at the actual point of waste generation^33^.

At the heart of the proposed theory lies the Moment-Based Precision Behavioral Fidelity (MBPBF) model, a conceptual construct that captures two essential dimensions of behavior. The first is habitual behavior, which reflects long-term adherence, routine compliance, and intrinsic motivation toward correct waste disposal practices^33^. The second is precision behavior, which assesses the accuracy and attentiveness involved in each act of bin usage, down to the micro-level of decision-making and execution^32^. Together, these dimensions feed into the foundational construct of PGSA (Point-of-Generation Segregation Accuracy), a shared element between both PGS Index and the Global Segregation Safety Scale (GSSS).

The PGS Index, though presently conceptual, distinguishes itself by incorporating Precision Behavior Analysis (PBA) and Staff Compliance Behavior (SCB) to construct a behavioral framework that holistically evaluates behavioral excellence in BMW segregation. In contrast, GSSS is an independent conceptual operational framework that incorporates PGSA data as one of its inputs but shifts the focus toward assessing procedural fidelity, system robustness, and compliance enforcement. Constructs such as Waste Quality Metrics (WQM), Standard Operating Procedure (SOP) compliance, and the Institutional Compliance Index (ICI) position it as inherently operational and system-oriented.

While the PGS Index remains behavior-centric, it still acknowledges systemic influences^32^, positioning itself as a hybrid index. GSSS, meanwhile, is rooted in procedural assessments, with behavioral inputs only indirectly shaping its outcomes. The only shared construct, PGSA, functions as the critical bridge linking behavioral precision to operational compliance.

A key innovation within the proposed Theory is the MBPBF model’s ability to capture behavioral subtleties. These include small but meaningful actions^32^, such as whether a healthcare worker hesitated before using a bin, self-corrected an error, or followed color-coded protocols through muscle memory. Such micro-behaviors^32,33^ are crucial in evaluating real-time segregation fidelity and are typically overlooked in conventional system-based audits.

Moreover, constructs such as Occupational Hazard Risk (OHR) and Environmental Contamination Potential (ECP) are reconceptualized through the PGST lens—not merely as technical failures, but as risk behaviors^32^. For instance, neglecting biohazard warnings or incorrectly disposing of sharps is no longer just procedural non-compliance; it becomes a quantifiable behavioral lapse with measurable risk and precision scores^32,33^.

By emphasizing behavioral science, the proposed PGST does not undermine systemic governance but enhances it. This becomes especially relevant in healthcare environments where institutional compliance may be high, yet behavioral lapses^32^ still lead to contamination events or safety breaches. Such lapses- like inappropriate bin usage, over-reliance on colored labels instead of content awareness, or skipping segregation steps often stem from training fatigue, insufficient reinforcement, or environmental misalignment (e.g., inconvenient bin placement)^33^. Over time, these evolve into behavioral drifts—repetitive deviations that become internalized, reducing long-term segregation fidelity and heightening occupational and environmental risks^32^.

In this context, the proposed conceptual PGS Index emerges as an illustrative differentiator. It captures the unseen, often intangible behaviors that shape actual waste segregation practices within clinical settings. By anchoring its evaluation in behavioral science while integrating systemic metrics^32^, PGST conceptually offers a more holistic and predictive model of waste governance. Ultimately, it bridges the gap between procedural compliance and human behavior, offering a theoretical pathway toward sustainable, high-fidelity biomedical waste segregation in healthcare systems.

**Limitations**

This study presents Bhadran’s Point-of-Generation Segregation Theory (PGST) as a conceptual and theoretical framework without real-world validation data at this stage. The proposed constructs and indices—PGSA (Point-of-Generation Segregation Accuracy), PBS (Precision Behavior Score), Point-of Generation Segregation Index (PGS Index), WQM (Waste Quality Metrics), and GSSS (Global Segregation Safety Scale)—have not yet been validated through empirical or longitudinal studies. Consequently, the findings should be interpreted as illustrative and exploratory rather than confirmatory. Moreover, the framework has not yet been evaluated across diverse healthcare contexts, and contextual factors such as infrastructure variability, cultural norms, and institutional policies may influence its applicability. The theoretical weighting schemes (α–ζ) used in the PGST Index are logically derived and will require empirical calibration in future studies. Therefore, multi-site, field-based validation and implementation research are recommended to assess the robustness, reliability, and practical utility of PGST in real-world healthcare environments.

**Conclusion**

The Point-of-Generation Segregation Theory (Bhadran’s PGST) emphasizes the pivotal role of individual healthcare professionals in the critical moments of waste generation. Despite established protocols and training, segregation errors often arise from cognitive overload, environmental distractions, and habitual behaviors at the clinical interface^34^. PGST conceptually proposes that many downstream segregation failures may originate upstream-during the split-second decision-making processes of healthcare professionals.

To address these challenges, healthcare facilities could consider targeted evidence-informed interventions:

- Real-time behavioral observations at high-risk generation points, such as operating theatres and isolation wards, to identify and correct segregation errors as they occur.
- Low-friction environmental design, including strategically placed color-coded bins and clear signage, to reduce cognitive load and facilitate correct segregation.
- Continuous behavioral reinforcement through micro-training sessions, visible nudges, and peer accountability systems to support sustained compliance^34^.
- Integration of PGST-aligned behavioral metrics into hospital quality dashboards, such as weekly PGSA scores for each ward, to monitor trends and incentivize improvements.

By embedding these strategies, PGST may help reframe biomedical waste management from a predominantly infrastructure-driven task into a behavior-oriented process. This theoretical framework conceptually aligns waste management with broader goals of hospital safety, infection prevention, environmental sustainability, and occupational health^34^.

**Future Research Opportunities**

The Point-of-Generation Segregation Theory (Bhadran’s PGST) provides a conceptual foundation that opens avenues for future interdisciplinary research aimed at empirical validation and model refinement:

- Empirical Validation: Multi-site observational studies and behavioral trials to test and validate PGST and PGSA metrics across varied healthcare contexts (urban/rural, public/private).
- Behavioral Drift Analysis: Longitudinal studies to track how training fatigue, cognitive load, and environmental changes impact behavioral drifts in waste segregation.
- AI-Driven Segregation Monitoring: Use of AI/computer vision tools to monitor bin-level behaviors and auto-flag behavioral lapses.
- Mental Models & Decision Trees: Understanding the mental schemas used by healthcare workers during waste disposal moments.
- Cross-Cultural Applicability: Studying how behavioral influences vary across healthcare cultures and regulatory environments.

**Productisation Opportunities**

- PGST frameworks may inform a range of practical solutions and scalable products:
- PGS Index Audit Toolkit: A standardized digital/physical toolkit for hospitals to measure PGSA scores and identify weak behavioral points.
- PGST Dashboard Module: Integration into hospital management software for potential real-time tracking of behavioral compliance at waste generation points.
- Behavioral Nudge Devices: Sensor-embedded color-coded bins that provide audio-visual cues when incorrect segregation is attempted.
- Training Simulators: Immersive VR/AR-based behavioral training modules simulating clinical waste disposal under stress or distraction.
- Accreditation Support: PGST-aligned certification support for NABH/JCI standards, tying waste segregation behavior to hospital excellence metrics.

**Concluding Perspective**

While PGST offers a novel perspective, it remains a theoretical and conceptual framework requiring further validation in diverse healthcare settings. Future studies should examine its applicability, assess its empirical reliability, and evaluate implementation feasibility. By shifting focus from policies to precision and from systems to individual behaviors, PGST may offer a structured foundation to enhance accountability and environmental stewardship

**Data Availability**

This manuscript does not report any data generation or analysis. Therefore, no datasets were created or analyzed for this study.

**Declarations**

- Ethics approval and consent to participate: **Not Applicable**
- Clinical Trial: **Not Applicable**
- Consent for Publication: **All authors have consented to the publication of this manuscript.**
- Competing Interests: **None declared**.
- Conflict of Interest: **None declared.**
- Funding: **Not Applicable**

**Master Table of Abbreviations, Full Forms & Constructs**

| Framework / Metric | Abbreviation | Full Form | Constructs / Elements |
| --- | --- | --- | --- |
| PGST | PGST | Point-of-Generation Segregation Theory | Core Behavioral Theory explaining segregation as a precision-based act  Behavioral constructs: Cognitive Anchoring (CA), Visual Discrimination (VD), Repetition Reinforcement (RR), Error Responsive Feedback (ERF).  Structural elements: PBS,PCS, MBPBF, PGSA, PGS Index, WQM, GSSS. |
|  |  |  |  |
|  |  |  |  |
|  |  |  |  |
|  |  |  |  |
|  |  |  |  |
|  |  |  |  |
| PGSA | PGSA | Point-of-Generation Segregation Accuracy | Elements: PBS (Precision Behavior Score) |
| PBS | PBS | Precision Behavior Score | Elements: Cognitive Anchoring (CA) – recalling & applying segregation rules; Visual Discrimination (VD) – instantly identifying correct bin; Repetition Reinforcement (RR) – repeating correct actions to form habits; Error Responsive Feedback (ERF) – self-correcting after mistakes |
| PCS | PCS | Precision Change Score | Elements: Measures change in PBS over time; Delta between baseline and follow-up PBS values |
| PGS Index | PGSI | Point-of-Generation Segregation Index | 6 Constructs: |
|  |  |  | 1. PGSA (Point-of-Generation Segregation Accuracy) |
|  |  |  | 2. OHR (Occupational Hazard Risk) |
|  |  |  | 3. ECP (Environmental Contamination Potential) |
|  |  |  | 4. ICI (Intra-facility Containment Integrity) |
|  |  |  | 5. SCB (Staff Compliance Behavior) |
|  |  |  | 6. TE (Training & Engagement) |
| MBPBF | MBPBF | Moment-Based Precision Behavioral Fidelity | PBS (Precision Behavior Score) |
|  |  |  | PCS (Precision Change Score) |
|  |  |  | PGSA(Point-of-Generation Segregation Accuracy) |
| GSSS | GSSS | Global Segregation Safety Scale | Core Element: Waste Quality Metrics (WQM), |
| WQM | WQM | Waste Quality Metrics | 6 Constructs: |
|  |  |  | 1. Segregation Accuracy Score (SA, PGSA-based) |
|  |  |  | 2. PPE Compliance (PPE) |
|  |  |  | 3. Spill Management (SM) |
|  |  |  | 4. Documentation Accuracy (DA) |
|  |  |  | 5. Temporary Storage Safety (TSS) |
|  |  |  | 6. Training & Awareness Maintenance (TE) |

References:

1. Chartier Y, Emmanuel J, Pieper U, Prüss A, Rushbrook P, Stringer R, editors. Safe Management of Wastes from Health-Care Activities. 2nd ed. Geneva, Switzerland: WHO Press; 2014. p. 1–146.
2. World Health Organization. Health-care waste [Internet]. Geneva: WHO; 2024 Oct 24 [cited 2025 Jul 3]. Available from:<https://www.who.int/news-room/fact-sheets/detail/health-care-waste>
3. Datta P, Mohi GK, Chander J. Biomedical waste management in India: critical appraisal. J Lab Physicians. 2018 Jan-Mar;10(1):6–14. doi:10.4103/JLP.JLP_89_17. PMID: 29403196; PMCID: PMC5784295.
4. Mondal, R. (2022). Impact of monitoring and training on biomedical waste segregation practice in hospitals. *International Journal Of Community Medicine And Public Health*, *9*(7), 2942–2944.<https://doi.org/10.18203/2394-6040.ijcmph20221763>
5. Mosquera M., Andrés‑Prado M. J., Rodríguez‑Caravaca G., Latasa P., Mosquera M.E. “Evaluation of an education and training intervention to reduce health care waste in a tertiary hospital in Spain.” *American Journal of Infection Control* 2014;42:894–897<https://www.researchgate.net/publication/262978164_Evaluation_of_an_education_and_training_intervention_to_reduce_health_care_waste_in_a_tertiary_hospital_in_Spain>
6. Bannour R., Ben Cheikh A., Bhiri S., Said Latiri H. *Impact of an educational training on healthcare waste management practices in a Tunisian university hospital (2019–2021)*. Antimicrobial Resistance & Infection Control. 2024;13:122<https://aricjournal.biomedcentral.com/articles/10.1186/s13756-024-01446-w?utm_source=chatgpt.com>
7. Central Pollution Control Board, Ministry of Environment, Forest & Climate Change. *Guidelines for Management of Healthcare Waste as per Biomedical Waste Management Rules, 2016*. Directorate General of Health Services, Ministry of Health & Family Welfare; 2018. Available from:<https://cpcb.nic.in/uploads/projects/bio-medical-waste/guidelines_healthcare_june_2018.pdf>
8. Patil GV, Pokhrel K. Biomedical solid waste management in an Indian hospital: a case study. Waste Manag. 2005;25(6):592-9. doi: 10.1016/j.wasman.2004.07.011. PMID: 15993343.
9. Gadicherla, S., Thapsey, H., Krishnappa, L., & Somanna, S. N. (2016). Evaluation of bio medical waste management practices in select health care facilities of Karnataka, India. *International Journal Of Community Medicine And Public Health*, *3*(10), 2722–2728.<https://doi.org/10.18203/2394-6040.ijcmph20163280>
10. Awodele O, Adewoye AA, Oparah AC. Assessment of medical waste management in seven hospitals in Lagos, Nigeria. BMC Public Health. 2016 Mar 15;16:269. doi: 10.1186/s12889-016-2916-1. PMID: 26979131; PMCID: PMC4791961.<https://pmc.ncbi.nlm.nih.gov/articles/PMC4791961/?utm_source=chatgpt.com>
11. Biniyam Sahiledengle (2019). *Self-reported healthcare waste segregation practice and its correlate among healthcare workers in hospitals of Southeast Ethiopia (Bale Zone).* BMC Health Services Research, 19:591.<https://bmchealthservres.biomedcentral.com/counter/pdf/10.1186/s12913-019-4439-9.pdf?utm_source=chatgpt.com>
12. Hamizah N. Mohamed, S. Khan & S. Jagtap (2024).
     "Waste 4.0: transforming medical waste management through digitalization and automated segregation." *Discover Sustainability* 5, 93.<https://link.springer.com/article/10.1007/s43621-024-00593-9>
13. Sepandi M, Alimohamadi Y, Afrashteh S, Rashti R. Occupational needle stick injuries and related factors among healthcare workers in military hospitals in Tehran. Nurs Open. 2023 Aug;10(8):5193-5201. doi: 10.1002/nop2.1755. Epub 2023 Apr 12. PMID: 37043412; PMCID: PMC10333905.
14. Amrita Vishwa Vidyapeetham. Amrita Hospital receives Best Environmental Practice Award – Kerala State Pollution Control Board [Internet]. Amrita Vishwa Vidyapeetham News. 2019 Jun 10 [cited 2025 Jul 3]. Available from:<https://www.amrita.edu/news/amrita-hospital-receives-best-environmental-practice-award-kerala-state-pollution-control-board/>
15. Evliya Felek, B.N., Karadağ Erkoç, S., Özçelik, M. *et al.* Impact of waste segregation training on medical and recyclable waste in an operating theater a quasi experimental study. *Sci Rep* **15**, 18430 (2025).<https://doi.org/10.1038/s41598-025-02797-z>
16. Bansod HS, Deshmukh P. Biomedical waste management and its importance: a systematic review. Cureus. 2023 Feb 3;15(2):e34589. doi:10.7759/cureus.34589. PMID: 36874306; PMCID: PMC9981497.
17. Amariglio A, Depaoli D. Waste management in an Italian Hospital's operating theatres: An observational study. Am J Infect Control. 2021 Feb;49(2):184-187. doi: 10.1016/j.ajic.2020.07.013. Epub 2020 Jul 11. PMID: 32663495.<https://pubmed.ncbi.nlm.nih.gov/32663495/>
18. Mugambe, R.K., Ssempebwa, J.C., Tumwesigye, N.M. *et al.* Healthcare waste management in Uganda: management and generation rates in public and private hospitals in Kampala. *J Public Health* 20, 245–251 (2012).<https://doi.org/10.1007/s10389-011-0459-6>
19. Qaiser S, Arif A, Quaid S, Ahsan T, Riaz K, Niaz S, Qureshi H, Ahmed W, Alam SE. Innovative solution to sharp waste management in a tertiary care hospital in Karachi, Pakistan. Infect Control Hosp Epidemiol. 2013 Dec;34(12):1297-305. doi: 10.1086/673978. Epub 2013 Oct 28. PMID: 24225615.<https://pubmed.ncbi.nlm.nih.gov/24225615/>
20. Mathur V, Dwivedi S, Hassan M, Misra R. Knowledge, Attitude, and Practices about Biomedical Waste Management among Healthcare Personnel: A Cross-sectional Study. Indian J Community Med. 2011 Apr;36(2):143-5. doi: 10.4103/0970-0218.84135. PMID: 21976801; PMCID: PMC3180941.
21. World Health Organization. Water and Sanitation for Health Facility Improvement Tool (WASH FIT): A practical guide for improving quality of care through water, sanitation and hygiene in health care facilities. Geneva: World Health Organization; 2017. Report No.: ISBN 978-92-4-151169-8. Updated cover and introduction in 2018.
22. Salia S. Sheriff, Abdulfatah Abdu Yusuf, Oluwole O. Akiyode, Ezekiel Fayiah Hallie, Saidi Odoma, Rebecca Alicia Yambasu, Kula Thompson-Williams, Charles Asumana, Sylvester Z. Gono, Mohammed A. Kamara,A comprehensive review on exposure to toxins and health risks from plastic waste: Challenges, mitigation measures, and policy interventions, Waste Management Bulletin, Volume 3, Issue 3, 2025, 100204, ISSN 2949-7507,<https://doi.org/10.1016/j.wmb.2025.100204>.
23. K.V. Sarma, K. Ramasubramanian, M.D. Srinivas, M.S. Sriram, Ganita-Yukti-Bhāṣā (Rationales in Mathematical Astronomy), Vol. I: Mathematics; Vol. II: Astronomy, Springer, 2008.
24. A.K. Bag, Madhava's Sine and Cosine Series, Indian National Science Academy, Park Street, Calcutta, 29 April 1975. Available at: <https://archive.org/details/madhavas/mode/2up>
25. K.V. Sarma, A History of the Kerala School of Mathematics, Vishveshvarananda Institute of Sanskrit and Indological Studies, 1972.
26. Pranav Prashant Dagwar, Deblina Dutta, Landfill leachate a potential challenge towards sustainable environmental management, Science of The Total Environment, Volume 926, 2024, 171668, ISSN 0048-9697,<https://doi.org/10.1016/j.scitotenv.2024.171668>.
27. Fick J, Söderström H, Lindberg R, Phan C, Tysklind M, Larsson J. Contamination of surface, ground, and drinking water from pharmaceutical production. Environ Toxicol Chem. 2009 Jun;28(12):2522–7. doi:10.1897/09-073.1.
28. Patil PM, Bohara RA. Nanoparticles impact in biomedical waste management. Waste Manag Res. 2020;38(11):1189–1203. doi:10.1177/0734242X20936761.
29. Krishnachandran, V. N. (2024). On Mādhava and his correction terms for the Mādhava–Leibniz series. arXiv preprint arXiv:2405.11134. <https://doi.org/10.48550/arXiv.2405.11134>
30. Patwary, M. A., O’Hare, W. T., & Sarker, M. H. (2011). Assessment of occupational and environmental safety associated with medical waste disposal in developing countries: a qualitative approach. Safety Science, 49(8), 1200–1207. https://doi.org/10.1016/j.ssci.2011.04.001
31. T.L. Tudor, C.L. Noonan, L.E.T. Jenkin,Healthcare waste management: a case study from the National Health Service in Cornwall, United Kingdom,Waste Management,Volume 25, Issue 6,2005,Pages 606-615,ISSN 0956-053X,https://doi.org/10.1016/j.wasman.2004.10.004.
32. Reason, J. (2000). Human error: Models and management. BMJ, 320(7237), 768–770. https://doi.org/10.1136/bmj.320.7237.768
33. Icek Ajzen, The theory of planned behavior, Organizational Behavior and Human Decision Processes,Volume 50, Issue 2,1991,Pages 179-211,ISSN 0749-5978, <https://doi.org/10.1016/0749-5978(91)90020-T>.
34. Gupta, D., Beniwal, P., Aggarwal, D., Gupta, P., Vikram, A., & Goyal, A. (2024). Biomedical waste management awareness and behaviour: A cross-sectional survey. International Journal of Medicine and Public Health, 14(4), 1298–1302. https://doi.org/10.70034/ijmedph.2024.4.237
