# Supplementary material for: Bhadran’s point of generation segregation theory for behavioral precision in biomedical waste management
Source: Sci Rep. 2025 Dec 16;16:2531. doi: 10.1038/s41598-025-32195-4 (PMC12820305; doi:10.1038/s41598-025-32195-4)
Supplement: Supplementary file 3 — Supplementary Material 3 [file 41598_2025_32195_MOESM3_ESM.docx]

**Response to Reviewers**

**General Statement**

We sincerely thank all reviewers for their constructive comments and thoughtful suggestions that have significantly improved the clarity, scientific rigor, and presentation quality of our manuscript titled “Bhadran’s Point-of-Generation Segregation Theory (PGST): A Novel Behavioral Framework and Metric System for Biomedical Waste Management.” All comments were carefully considered and addressed in the revised version. Below, we provide a detailed point-by-point response outlining the actions taken.

**Reviewer 1**

**Comment:** This work proposed a hybrid behavioral systems model designed to measure, benchmark, and improve BMW management. The content is clear, the results are sufficient, and the logic is strong. To sum up, I agree that it can be accepted.

**Response:** We thank Reviewer 1 for the positive assessment and for recognizing the clarity, logical structure, and potential applicability of the proposed PGST framework. We have nonetheless refined the manuscript for enhanced consistency, formatting, and alignment with journal standards.

**Reviewer 2**

We sincerely thank Reviewer 2 for their insightful and constructive feedback, which has been invaluable in improving the scientific precision and presentation of this manuscript. Detailed responses are as follows:

**Comments:**

1. **Clarify the research scope and validation status**: “Please state explicitly in both the abstract and the introduction that PGST and the associated indices (PBS, PGSA, WQM, GSSS) are theoretical constructs not yet validated through empirical data. Phrases that imply universal or proven effectiveness should be reworded as “proposed,” “conceptual,” or “illustrative.” This will align expectations and maintain scientific precision”.

**Response:** In response, we have revised both the Abstract and Introduction to clearly state that Bhadran’s Point-of-Generation Segregation Theory (PGST) and its associated indices—Precision Behavior Score (PBS), Point-of-Generation Segregation Accuracy (PGSA), Waste Quality Metrics (WQM), and Global Segregation Safety Scale (GSSS)—are conceptual and theoretical constructs that have not yet undergone empirical validation.

All statements that previously implied established or universal effectiveness have been carefully reworded to use terms such as “proposed,” “conceptual,” “illustrative,” or “theoretical.” This ensures scientific precision and appropriately aligns reader expectations with the current developmental stage of the framework.

2. **Improve methodological transparency**: A short “Methodology” or “Framework Derivation” subsection should explain:
• How variables, coefficients, and weights were determined.
• Whether data in Tables 1–2 were taken directly from literature, re-coded, or simulated.
• The basis for assigning the P/N/B ratings and how they feed into the PGSA and WQM equations.
This information is essential for readers to reproduce or verify the logic of the framework.

**Response:** We thank the reviewer for this valuable suggestion to enhance methodological clarity. A new subsection titled “Framework Derivation and Methodological Basis” has been added to the revised manuscript. This section now explicitly describes:

- The data source and derivation of Tables 1–2, confirming that all entries were directly extracted and standardized from published literature rather than simulated.
- The theoretical logic and normalization process used to determine the weighting coefficients (α–ζ) across the six constructs.
- The criteria for assigning Positive (P), Negative (N), and Borderline (B) ratings based on construct performance, and their subsequent integration into the Point-of-Generation Segregation Accuracy (PGSA) and Waste Quality Metric (WQM) equations.

This addition improves the framework’s transparency, reproducibility, and interpretive validity, aligning with the reviewer’s recommendation.

3. **Ensure that formulas and examples are fully interpretable:** Each formula should have clearly defined symbols, units, and value ranges. Please include a concise notation table summarizing all symbols (α, β, γ, δ, ε, ζ etc.) and their meaning. In the worked examples, report numerical results directly in the text (e.g., “PGSA = 7.5 produced WQM 1 = 15 points”) so that the reader can follow the derivation step by step.

**Response:** We appreciate the reviewer’s insightful suggestion to improve clarity and interpretability. In response, the following revisions have been made:

- A comprehensive notation table has been added (Table ___) summarizing all symbols, coefficients (α–ζ), and corresponding units or value ranges.
- Each formula has been revised to ensure all variables and parameters are explicitly defined at first mention.
- The worked examples now include numerical outputs directly within the text to demonstrate how PGSA and WQM values are derived step by step.

These enhancements collectively improve transparency and ensure that readers can easily replicate and understand the computation process.

4. **Align conclusions with the evidence presented:**The conclusions currently over-state the level of empirical verification. Revise to focus on what the current data and examples show, not on what they imply or might demonstrate after validation.

**Response:** We thank the reviewer for this important observation. The Conclusion section has been carefully revised to better reflect the conceptual and theoretical nature of the study. All statements implying empirical validation or proven applicability have been rephrased to emphasize that:

- The PGST framework and its associated indices (PBS, PGSA, WQM, GSSS) are proposed theoretical models;
- The illustrative examples serve only to demonstrate internal logic and potential applicability;
- Future work will focus on empirical validation across diverse healthcare and waste-management settings.

These revisions ensure that the conclusions accurately align with the current conceptual stage of the research and maintain scientific precision.

5. **Add an explicit limitations paragraph:** Include a short paragraph—either in the Discussion or before the Conclusions—acknowledging the main boundaries of the study:

• Conceptual stage without field validation.
• Reliance on theoretical weighting schemes.
• Possible cultural or institutional variability in behavioral indicators.

This will strengthen transparency and credibility.

**Response:** We thank the reviewer for this constructive suggestion. An explicit Limitations paragraph has now been added before the Conclusion section. The new paragraph clearly states that:

- The PGST framework and its indices are currently in the conceptual stage without field validation;
- The weighting coefficients (α–ζ) are theoretical and require empirical calibration;
- The applicability of behavioral indicators may vary across different institutional, cultural, and infrastructural contexts.

This addition strengthens the transparency and scientific credibility of the manuscript by clarifying the scope and boundaries of the present work.

6. **Presentation and formatting:**

• Verify equation numbering, consistent font and alignment.
• Place table captions above tables and figure captions below figures.
• Shorten historical context sections slightly to maintain focus on biomedical applications.
• Review references for duplication and ensure all DOIs are active.

**Response:** We appreciate the reviewer’s detailed feedback on presentation and formatting. The following revisions have been implemented to improve overall clarity and consistency:

- Equation numbering, font, and alignment have been thoroughly reviewed and standardized across the manuscript.
- Table captions have been repositioned above each table, and figure captions are now placed below each figure, following journal formatting guidelines.
- The historical background section has been shortened to enhance focus on the biomedical and behavioral applications of PGST.
- The reference list has been carefully checked to remove any duplicate entries, and all DOIs have been verified for accuracy and functionality.

These refinements ensure the manuscript meets formatting and readability standards for publication.

**Concluding Statement**

We thank both reviewers for their time, expertise, and valuable insights. All comments have been addressed comprehensively and to the level of expectation. The revised manuscript reflects these improvements in structure, precision, and presentation, and we believe it now meets the standards required for publication.
